# Investigation of the Suitability of the ROTEM Assay to Measure Coagulation Potential in Blood From Patients on Concizumab Prophylaxis

**DOI:** 10.1111/hae.70203

**Published:** 2026-01-30

**Authors:** Hermann Eichler, Nora V. Butta, Anne Riddell, Cecilia Augustsson, Marianne Kjalke, Kasper Jensen, Andrea Paramo‐Florencio, Jan Astermark, Pratima Chowdary, Victor Jiménez‐Yuste

**Affiliations:** ^1^ Institute of Clinical Haemostaseology and Transfusion Medicine Saarland University and Saarland University Hospital Homburg Germany; ^2^ Department of Haematology La Paz University Hospital, Coagulopathies and Disorders of Haemostasis Group IdiPaz Madrid Spain; ^3^ KD Haemophilia and Thrombosis Centre Royal Free Hospital London UK; ^4^ Health Services Laboratory London UK; ^5^ Department of Clinical Chemistry and Pharmacology University and Regional Laboratories Region Skåne Malmö Sweden; ^6^ Novo Nordisk A/S Måløv Denmark; ^7^ Novo Nordisk A/S Aalborg Denmark; ^8^ Centre for Thrombosis and Haemostasis Skåne University Hospital Malmö Sweden; ^9^ Department of Haematology Cancer Institute University College London London UK; ^10^ Department of Haematology La Paz University Hospital Coagulopathies and Disorders of Haemostasis Group IdiPaz Autónoma University Madrid Spain

**Keywords:** blood coagulation tests, concizumab, haemophilia A, haemophilia B, haemostasis, thromboelastometry

## Abstract

**Background:**

Rotational thromboelastometry (ROTEM) aims to measure the coagulation potential in whole blood. Concizumab, an anti‐tissue factor pathway inhibitor (TFPI) antibody for prophylaxis in haemophilia, enhances tissue factor (TF)‐initiated coagulation by preventing inhibition of activated factor X (FXa), thus increasing thrombin generation.

**Objectives:**

To evaluate a modified ROTEM assay for monitoring patients on concizumab prophylaxis.

**Methods:**

The TF reagent (r_exTEM) was diluted 50,000‐fold to make the ROTEM assay sensitive to haemophilia and to concizumab. The effect of concizumab was evaluated in the modified ROTEM in haemophilia A (HA)‐like blood (normal blood with added anti‐FVIII antibody). ROTEM analysis was performed in blood from patients participating in the explorer7/8 trials during 24 weeks of concizumab prophylaxis. Rotrol N plasma was used as quality control.

**Results:**

In vitro experiments showed concizumab concentration‐dependent reduction in clot time (CT) and increase in clot development (α‐angle) in HA‐like blood. At three of four clinical sites, CT and clot development were stable, variance of the control plasma was ≤12.4% and TF content of the diluted reagent (r_exTEM) was consistent. At these three sites, the correlation between CT versus concizumab exposure, free TFPI and thrombin generation assay parameters was weak (‐0.508 to +0.359). Prothrombin time positively correlated with CT (0.523) and negatively correlated with α‐angle (‐0.659).

**Conclusion:**

Due to the poor correlation between ROTEM parameters, concizumab exposure, free TFPI and thrombin generation parameters and the lack of consistent and reliable performance of the modified ROTEM assay, it cannot be recommended for general monitoring of patients on concizumab prophylaxis.

## 1 Introduction

Haemophilia A (HA) and haemophilia B (HB) are inherited X‐linked bleeding disorders caused by the absence of factor (F) VIII and FIX, respectively [1]. The lack of these coagulation factors leads to reduced coagulation potential and, in turn, prolonged and spontaneous bleeding [1]. There is increasing interest in monitoring the coagulation potential in patients with inherited coagulopathies, including haemophilia, to optimise and tailor treatment [2]. Recently, novel treatment options became available that warrant investigation of global assays to measure their efficacy [3, 4].

Rotational thromboelastometry (ROTEM) is a whole blood viscoelastic assay that measures changes in clot tensile strength due to fibrin polymerisation over time. As a point‐—of‐care test, it has been found to be valuable in monitoring changes in the context of acute bleeding, where it appears to be sensitive to low platelet levels, low fibrinogen and low clotting factors [5]. During ROTEM, a pin rotates in an anticoagulated whole blood sample, and as coagulation is initiated with calcium and a trigger, clot formation increases resistance, which is recorded graphically and numerically. ROTEM measures the time it takes for the clot to appear (clot time, CT), the time from initiation of clotting until a predefined clot firmness is reached (clot formation time, CFT), the rate of clot development (α‐angle), as well as the strength of the clot (maximal clot firmness) [5].

The Scientific and Standardization Committee (SSC) of the International Society of Thrombosis and Hemostasis (ISTH) has recommended intrinsic pathway activation for routine clinical monitoring of haemophilia using ROTEM or thromboelastography [6]. The standard ROTEM assay initiated with tissue factor (TF) is not sensitive to the lack of FVIII or FIX in haemophilic blood due to the high concentration of TF in the reagent (r_exTEM) used for activation [7, 8, 9]. As there is no reliable and standardised source of extrinsic pathway activator with a minute amount of TF, the working group was not able to provide a recommendation for extrinsic pathway activation in haemophilic blood samples [6].

Concizumab is a humanised monoclonal antibody that binds the K2 domain of TF pathway inhibitor (TFPI) and prevents TFPI's inhibition of activated FX (FXa). The reduced activity of TFPI allows sufficient activity of FXa to ensure thrombin generation in the absence of FVIII and FIX [10]. Concizumab is approved by the European Medicines Agency [8], the U.S Food and Drug Administration [9] and in other countries for once‐daily, subcutaneous prophylaxis in HA or HB with inhibitors (HAwI/HBwI) and without.

As concizumab enhances the TF‐mediated initiation phase of coagulation, its function can be assessed using TF‐initiated assays. Previously, diluted TF‐containing prothrombin time (PT) assay reagents (e.g., Innovin [Siemens Healthcare] or HemosIL Recombiplastin 2G [Werfen]) have been used in thromboelastography to evaluate the effect of concizumab and other anti‐TFPI antibodies such as befovacimab), as well as recombinant activated FVII (rFVIIa) and rFVIIa variants [10, 11, 12, 13, 14, 15]. The aim of the present study was to evaluate a modified ROTEM assay with diluted TF reagent (r_exTEM) to measure the coagulation potential of blood of patients on concizumab prophylaxis.

## 2 Methods

### 2.1 Donors

Peripheral blood was drawn from healthy volunteers who were members of the Danish National Corps of Voluntary Blood Donors (approved by “De videnskabsetiske komiteer for region hovedstaden”; VEK journal no. H‐D‐2007‐0055). All donors were 18 years or older and they had not taken acetyl salicylic acid for 10 days or other non‐steroidal anti‐inflammatory drugs for 72 h prior to blood sampling. Blood was drawn into 3.2% citrated vacutainers (BD), and the first 2 mL were discarded.

### 2.2 Modified ROTEM and Analysis

For the modified ROTEM assay, a 2941‐fold predilution of the TF reagent (r_exTEM, TEM Innovations GmbH) in 20 mM 4‐(2‐hydroxyethyl)‐1‐piperazineethanesulfonic acid (HEPES), 140 mM NaCl, pH 7.4 and 2% bovine serum albumin (BSA) was prepared resulting in a 50,000‐fold dilution. The diluted TF reagent (20 µL) was added to the ROTEM sample cup before adding 20 µL calcium reagent (star‐tem, TEM Innovations GmbH) and 300 µL blood. HA‐like whole blood was prepared 30 min after blood sampling by incubating normal donor blood with a neutralising anti‐FVIII polyclonal antibody (Sheep anti‐Human Factor VIII without glycerol, Prolytix); untreated donor blood was used as normal control. For ROTEM in vitro experiments, concizumab was added to the HA‐like whole blood at plasma concentrations between 10 and 150,000 ng/mL, assuming a haematocrit of 50%.

**FIGURE 1:**
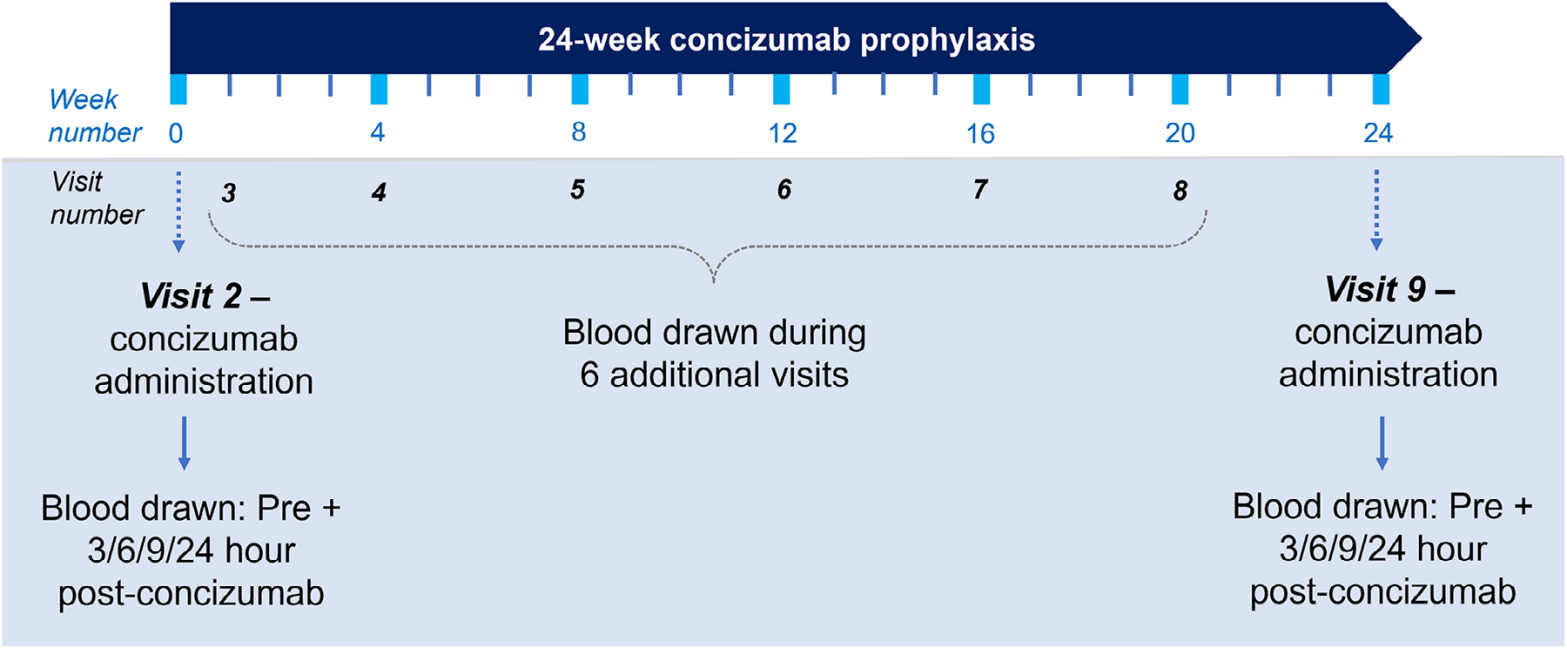
Timeline of study visits and blood sampling from patients participating in the phase 3 explorer 7/8 trials during 24 weeks of concizumab prophylaxis.

The modified ROTEM was performed in duplicate on citrated blood samples from 12 patients participating in the explorer7 and explorer8 phase 3 trials (ClinicalTrials.gov identifiers: NCT04083781, NCT04082429; Figure 1, Table 1). Patients included into the explorer7 and 8 trials were male, aged ≥12 years and weighing more than 25 kg, with HA or HB with and without inhibitors respectively [16, 17]. All patients were either concizumab‐naïve or had been off concizumab prophylaxis for at least 5 months when the first samples were taken at visit 2. ROTEM analysis was performed within 30 min to 2 h after blood sampling. Quality control samples included Rotrol N plasma (TEM Innovations GmbH) analysed both with diluted and the undiluted TF reagent in order to analyse inter‐ and intra‐site variability. After study completion, the diluted TF reagent of each single ROTEM measure was frozen, shipped and analysed in batch for TF content using a chromogenic FX activation assay. The diluted TF sample was added to 0.1 nM rFVIIa (Novo Nordisk) and 175 nM FX (Prolytix) in 50 mM HEPES, 100 mM NaCl, 10 mM CaCl_2_, 0.1% PEG8000, 1 mg/mL BSA, pH 7.3, followed by 5 min incubation at room temperature. The reaction was stopped by adding half a volume 20 mM EDTA in 50 mM HEPES, 100 mM NaCl, pH 7.3. Activity was measured for 10 min at 405 nm after adding 0.5 mM FXa chromogenic substrate (S2765, Chromogenix). The activity was converted to TF concentration by using a calibration curve of recombinant lipidated TF_1‐244_ (Novo Nordisk). Measurement of concizumab level using enzyme‐linked immunosorbent assay (ELISA), free TFPI (TFPI not in complex with concizumab measured using Asserachrom Total TFPI ELISA [Diagnostica Stago]) and thrombin‐generation assays (calibrated automated thrombogram initiated with TF‐containing reagent [PPP‐Reagent low, 1 pM TF; Thrombinoscope BV, Stago) was performed at the central laboratory measuring all clinical samples from explorer7 and explorer8, as described previously [18].

**TABLE 1:** Patients in ROTEM study per participating clinical site (A–D).

Site	A	B	C	D
Number of patients included				
HA	1	1	1	4
HB	—	—	1	2
HBwI	2	—	—	—
Comments	The two HBwI patients were included at visit 5^a^		The HB patient discontinued participation after visit 2^a^	

Abbreviations: HA, haemophilia A; HB, haemophilia B; HBwI, haemophilia B with inhibitors.

^a^ Study visits with blood sampling during the explorer7 and explorer8 phase 3 trials included visit 2 (before starting concizumab dosing) and monthly visits during 24 weeks of concizumab prophylaxis. For a timeline, please see Figure 1.

### 2.3 Statistical Analysis

ROTEM parameters in patient samples were compared by Spearman's correlation coefficients with concizumab plasma levels (exposure), free TFPI, thrombin generation parameters (peak height, endogenous thrombin potential [ETP], velocity index [maximal rate of thrombin generation]), and other laboratory parameters, including PT. A correlation coefficient larger than +0.6 or smaller than ‐0.6 was considered a fair correlation.

## 3 Results

### 3.1 Establishing a ROTEM Assay With Diluted TF Reagent

**FIGURE 2:**
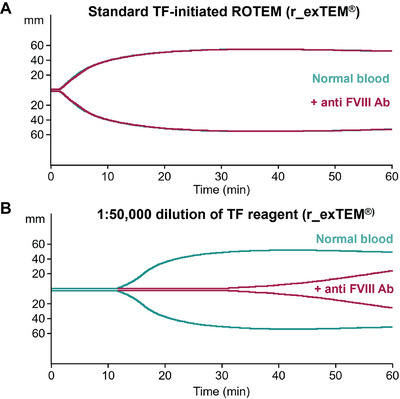
Development of a ROTEM method sensitive to haemophilia conditions and the in vitro effect of concizumab. Standard ROTEM using normal TF reagent (r_exTEM) to initiate coagulation is not sensitive to the lack of FVIII in an antibody‐induced mimic of haemophilic blood (A). In the modified ROTEM assay, sensitivity to reduced FVIII was increased by diluting the TF reagent 50,000‐fold, resulting in a TF concentration of 0.1 pM (B). Ab, antibody; FVIII, factor VIII; ROTEM, rotational thromboelastometry; TF, tissue factor.

**FIGURE 3:**
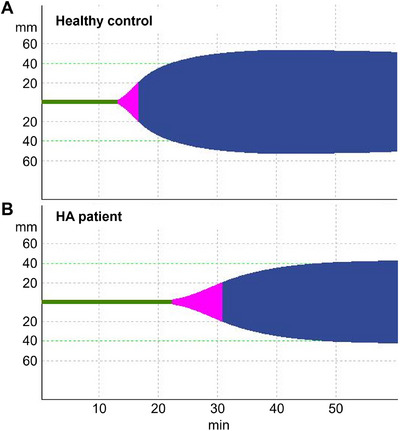
ROTEM profiles of blood samples from a healthy control and a patient with haemophilia A (HA). r_exTEM containing 50,000‐fold diluted TF reagent was used to initiate coagulation in (A) blood from a healthy control and (B) in blood from a patient with HA (bottom, residual FVIII level of 6%). HA, haemophilia A; TF, tissue factor.

A ROTEM method sensitive to haemophilia conditions and to the in vitro effect of concizumab was developed. The EXTEM method initiated with a relatively high TF concentration (approximately 2 nM) did not discriminate between normal blood and blood with a neutralising anti‐FVIII antibody to mimic HA (Figure 2A). However, when the TF reagent (r_exTEM) was diluted 50,000‐fold, resulting in an approximate plasma concentration of 0.1 pM, the clot formed later in the HA‐like blood than in normal blood and the clot formation rate was reduced (Figure 2B). When the method was applied to HA patient blood with mean residual FVIII of 5.9% (standard deviation [SD] 6.5%), the clot formation was delayed compared with healthy control blood (Figure 3). In the healthy control versus HA whole blood samples, the clot time was 763 s (SD 21.2) versus 1393.5 s (SD 122.4), respectively, while the clot development rate (α‐angle) was 53° (SD 1.4) versus 31° (SD 5.5), respectively. These results are in agreement with previous results [19] and confirm that ROTEM with diluted TF reagent can distinguish between normal and haemophilic blood.

**FIGURE 4:**
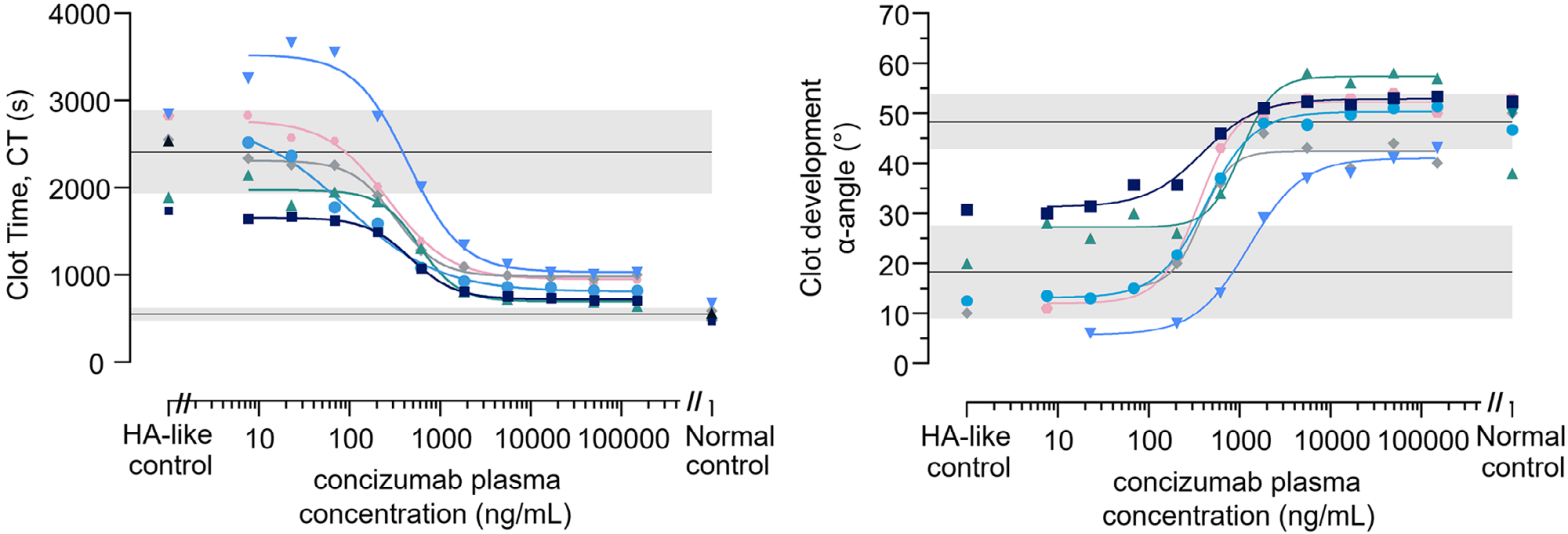
In vitro spiking of concizumab into HA‐like blood from healthy donors with a neutralising FVIII antibody. ROTEM was measured using TF reagent (r_exTEM) diluted 50,000‐fold, and clot time (CT) and clot development (α‐angle) are shown for six individual donors. Normal control corresponds to samples without the FVIII antibody added. The black line and grey‐shaded areas correspond to mean ± 1 SD of HA‐like control and normal blood. HA, haemophilia A; SD, standard deviation.

The effect of concizumab in the modified ROTEM assay was evaluated in spiking experiments using HA‐like blood. When concizumab was added to HA‐like blood (*n* = 6), a concentration‐dependent reduction of clot time and an increase in clot development rate (α‐angle) was observed (Figure 4). The Spearman's correlation between concizumab levels and ROTEM clot time or α‐angle was ‐0.863 and 0.809, respectively, demonstrating strong correlation between increased concentration of concizumab and a shortening of the clot time (negative correlation) and an increase in rate of clot development (positive correlation).

### 3.2 Application of ROTEM Assay With Diluted TF Reagent at Clinical Sites

**FIGURE 5:**
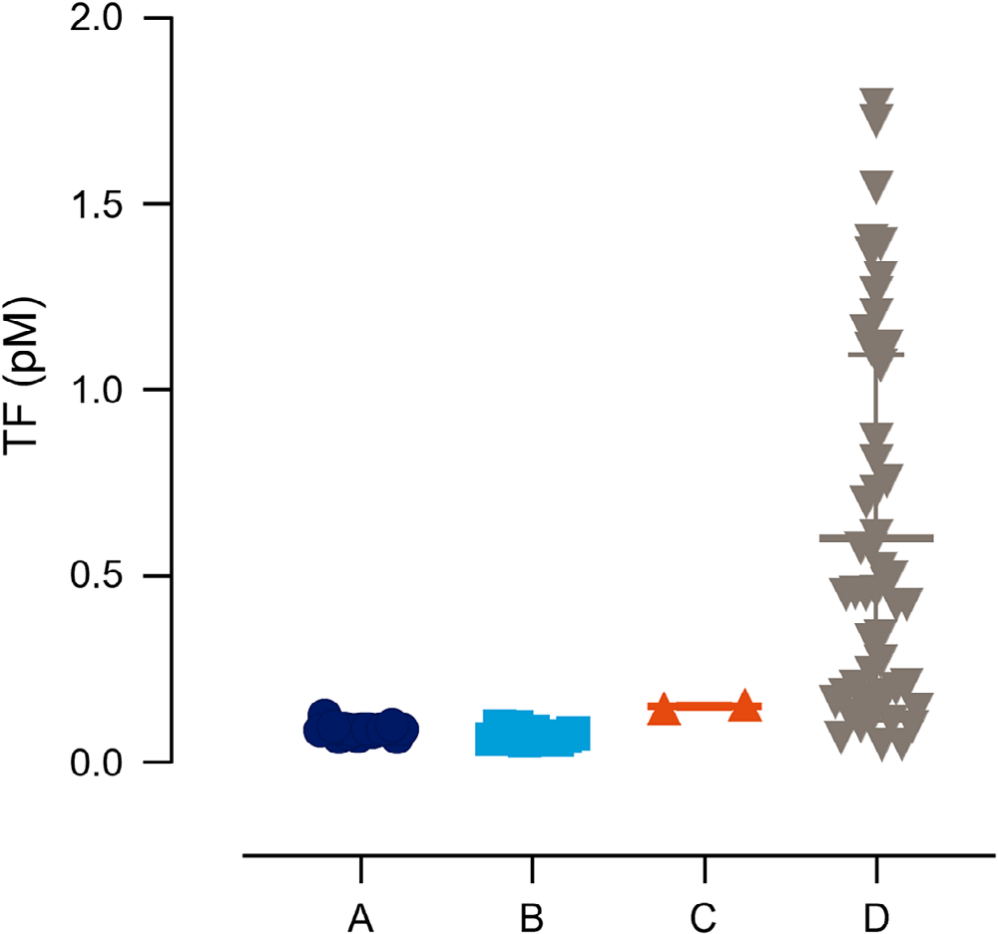
TF content in diluted TF reagent (r_exTEM) as measured in a TF/FVIIa‐mediated FX activation assay. FVIIa, activated factor VII; FX, factor X; TF, tissue factor.

Twelve patients participating in the explorer7 and explorer8 phase 3 trials (Table 1) had their blood samples analysed by the modified ROTEM assay before starting concizumab dosing (visit 2) and at monthly visits during 24 weeks of concizumab prophylaxis (Figure 1). Control experiments using Rotrol N plasma were performed in parallel with the analysis of patient samples. At sites A–C, the coefficient of variation (CV) of clot time of the Rotrol N plasma analysed with diluted TF reagent was ≤12.4% (Table 2). At site D, the corresponding CV was 35.4%. Analysis of the TF activity in the diluted TF reagent used at the four sites revealed that the TF content was consistent between sites A–C but was increased and more variable at site D (Figure 5).

**TABLE 2:** Clot time values of TF reagent (r_exTEM) diluted 1:50,000 at the four participating clinical sites in the study (A–D).

Site	A	B	C	D
Number of measurements	26	18	15	76
Mean CT (s)	905	872	993	830
SD (s)	112	67	119	294
CV (%)	12.4	7.7	12.0	35.4

Abbreviations: CT, clot time; CV, coefficient of variation; SD; standard deviation; TF, tissue factor.

**FIGURE 6:**
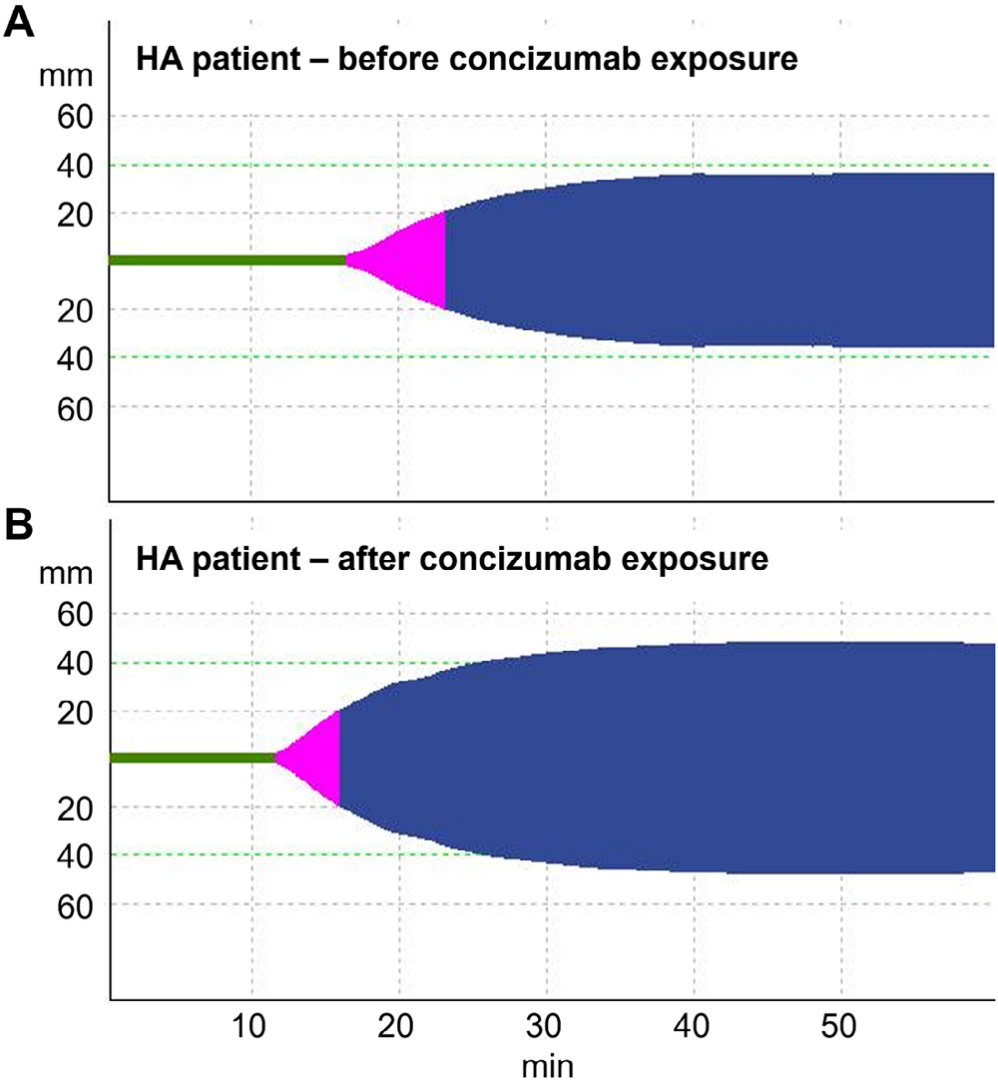
ROTEM profiles of blood samples from a patient with haemophilia A (HA) before and after receiving concizumab prophylaxis. r_exTEM containing 50,000‐fold diluted TF reagent was used to initiate coagulation in blood from a patient with HA (A) before and (B) after concizumab treatment. HA, haemophilia A; TF, tissue factor.

**FIGURE 7:**
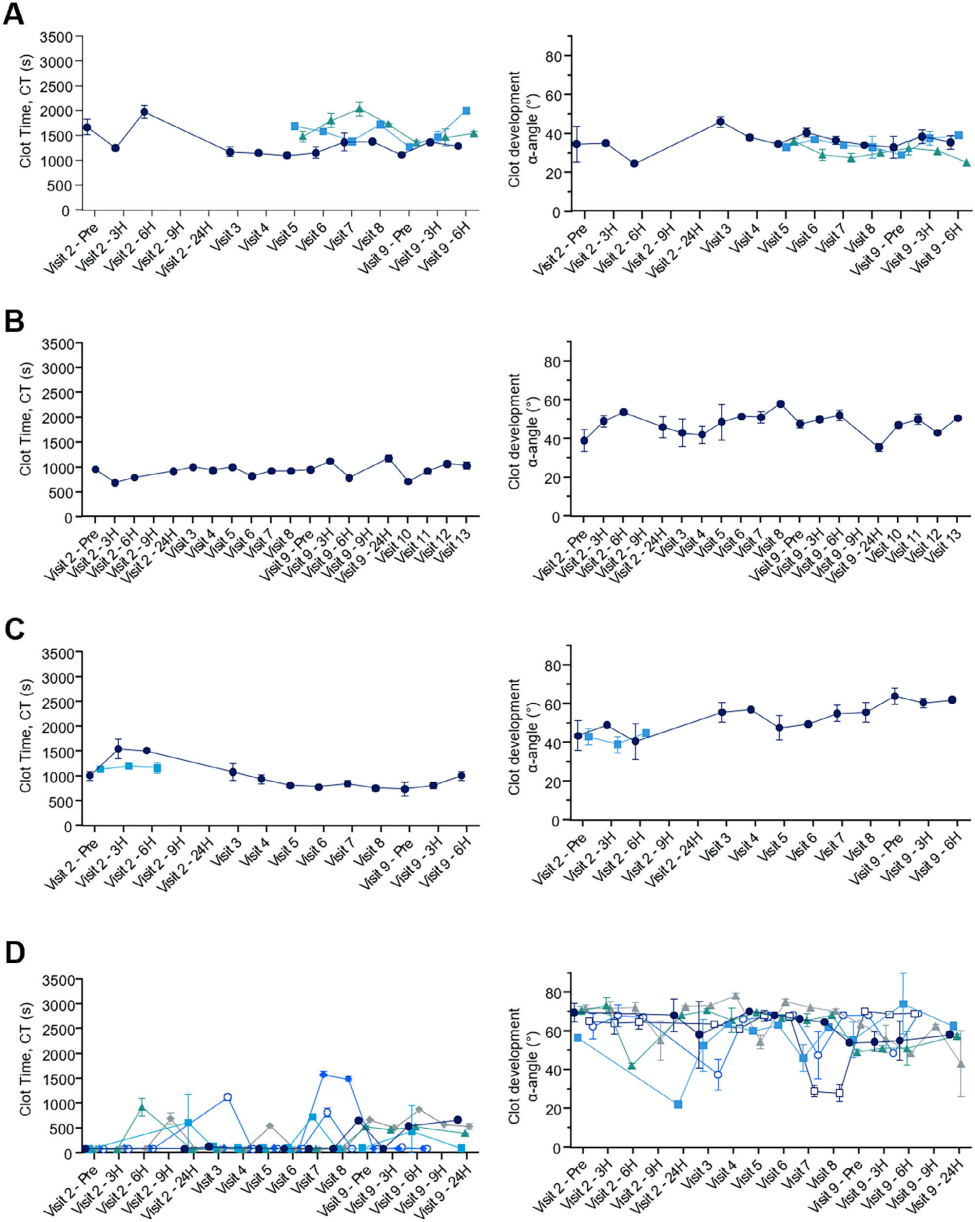
Individual haemophilia patients’ ROTEM clot time and clot development (α‑angle) at hospital visits at the four participating sites over 24 weeks. Blood was drawn at the four participating sites (panels A–D) from patients before concizumab administrations at visit 2 and 9 (‘Visit 2 – Pre’ and ‘Visit 9 – Pre’), 3, 6 and 24h after concizumab administration, as well as during visits in‐between (visits 3–8). At site B, samples were taken up until visit 13. Each line represents one patient. CT, clot time.

An example of a patient ROTEM trace at baseline and at concizumab steady state is shown in Figure 6. After concizumab treatment, the delay in clot formation observed in HA blood was mostly abrogated and the rate of clot development improved. Overall, the clot time and clot development (α‐angle) values were stable at sites A–C, while higher variability and shorter clot time values were seen at site D (Figure 7). The variation in TF content at site D suggests that the higher variability observed was, at least in part, due to dilution imprecision of the TF reagent. Due to the high variability in the clot time of both the Rotrol N plasma and the patient samples and the variability of TF content in the diluted TF reagent, data from site D were excluded from further analyses.

The correlation between ROTEM parameters and concizumab exposure, free TFPI (i.e., TFPI not in complex with concizumab) and thrombin generation parameters was analysed to evaluate the reliability of the modified ROTEM assay to monitor coagulation during concizumab exposure (Table 3). For data obtained at sites A–C (*n* = 6 patients, 59 datapoints), a longer ROTEM clot time was observed with increased concizumab exposure, seen as a weak positive correlation (+0.359). This is opposite to what would be expected, as increased concizumab exposure would provide a more procoagulant state and hence a reduced clot time. Likewise, the ROTEM clot time increased with decreased level of free TFPI, seen as a weak negative correlation of ‐0.313. A reduction of free TFPI by concizumab and thereby increased FXa activity would lead to a more procoagulant state, and a concomitant shortening of the ROTEM clot time was expected. The ROTEM CFT showed similar, unexpected results as the ROTEM clot time, that is, longer CFT with increased concizumab exposure (weak positive correlation of +0.415) and longer CFT with reduced free TFPI (weak correlation of ‐0.336). The rate of clot development (α‐angle) was expected to increase with increased concizumab exposure and decrease with increased free TFPI, but the observed correlations were again opposite to those expected, that is, a weak negative correlation of ‐0.435 for concizumab exposure and a weak positive correlation of +0.351 for free TFPI were obtained.

**TABLE 3:** Spearman's correlation coefficients between ROTEM parameters and plasma analysis results at sites A–C.

			Thrombin generation parameters *n* = 46			
	Concizumab plasma levels (exposure) *n* = 59	Free TFPI *n* = 58	Thrombin peak	Rate (velocity index)	ETP	PT (*n* = 44)
Clot time	0.359	−0.313	−0.238	−0.166	−0.508	0.523
Clot formation time	0.415	−0.336	−0.153	−0.061	−0.461	0.598
Rate of clot development (α‐angle)	−0.435	0.351	0.127	0.035	0.443	−0.659
Maximal clot firmness	−0.017	−0.114	−0.245	−0.330	0.064	−0.368

Abbreviations: ETP, endogenous thrombin potential, i.e., area under the thrombin generation curve; PT, prothrombin time; ROTEM, rotational thromboelastometry; TFPI, tissue factor pathway inhibitor.

Next, the ROTEM assay results were compared with the results from the thrombin generation assay (*n* = 6 patients, 46 datapoints). The ROTEM parameters followed the results from the thrombin generation assay in the expected direction: ROTEM clot time was reduced when the rate of thrombin generation (velocity index) was increased (weak negative correlation of ‐0.166), the thrombin peak was increased (weak negative correlation of ‐0.238) and the ETP was increased (negative correlation of ‐0.508). The correlation coefficients were similar for other ROTEM parameters, such as CFT (weak negative correlation) and α‐angle (weak positive correlations) (Table 3). Interestingly, when the ROTEM values were compared to other values from the central laboratory, the PT showed a higher positive correlation with the ROTEM clot time (+0.523) and the CFT (+0.598) and a negative correlation with the α‐angle (‐0.659) (Table 3). These correlations were all in the directions expected, that is, a short PT is indicative of a procoagulant state which is also reflected in the decreased values for clot time and CFT, and an increased value for clot development (α‐angle).

### 3.3 Bleeding Events

There were only two bleeding events reported for sites A‐C during this study; one mild‐to‐moderate traumatic bleed in a HA patient (Figure 7B; bleeding recorded one day prior to visit 9) and one severe spontaneous bleed in a HB patient (Figure 7C; bleeding reported one day after visit 2, and the patient discontinued concizumab prophylaxis). No apparent changes in ROTEM parameters were visible at the visits closest to the bleeding events.

## 4 Discussion

The aim of the present study was to evaluate the use of a modified, TF‐induced ROTEM assay sensitive to low FVIII and to concizumab to monitor the coagulation potential of patients on concizumab prophylaxis. This was achieved by diluting the TF reagent (r_exTEM) 50,000‐fold, resulting in a TF concentration of 0.1 pM. Using this diluted TF reagent, a strong correlation between concentrations of concizumab added to normal blood with an antibody‐induced mimic of HA and the ROTEM clot time and clot development (α‐angle) values was observed (Figure 4). Increased concizumab concentrations resulted in a shortening of the ROTEM clot time (negative correlation) and an increase in α‐angle (positive correlation), demonstrating haemostatic potential for concizumab in HA‐like blood.

When this ROTEM method was applied at four clinical sites participating in the phase 3 studies for concizumab, three were able to obtain stable ROTEM clot time and clot development (α‐angle) values in patient whole blood samples and reproducible TF content in the diluted TF reagent (Figures 5, 7). For these three sites, mostly weak correlations were observed between ROTEM clot time and clot development (α‐angle) values and plasma analysis parameters, such as concizumab exposure, free TFPI and parameters from the thrombin generation assay (Table 3). Notably, the correlations between the ROTEM parameters and concizumab exposure and free TFPI were not in the direction expected. The correlation between ROTEM clot time and thrombin generation assay parameters was weak, but in the directions expected. These results contrast with our initial spiking experiments where the correlations were stronger, and in the directions expected. It should be noted that in the spiking experiment, concizumab concentrations below the exposure level of concizumab in the clinical trials were included. The weak correlation between ROTEM parameters and concizumab exposure in patient samples could be a result of the patients being on steady‐state concizumab prophylaxis during most of the time in the study. Only four patients at sites A–C had their baseline samples prior to concizumab exposure analysed by ROTEM, but no unambiguous improvement of the ROTEM parameters was seen between their first analysis (visit 2 Pre, Figure 1) and the following analyses performed at time points after concizumab administration. The most probable explanation for the results reported here is that the ROTEM parameters are influenced by factors other than TFPI inhibition, and the assay could be more sensitive to differences in coagulation factor concentrations than to small fluctuations of concizumab at steady state. This notion is supported by the fair correlation observed between ROTEM clot time and PT, which is sensitive to the level of fibrinogen, prothrombin, FV, FVII and FX. A previous study showed that prothrombin level at the high end of the normal range and FV at the low end of the normal range increase thrombin generation in HA [20]. It is likely that such changes in factor levels in the patient samples also influence the ROTEM results.

It is conceivable that the variations in TF content may have led to the observed variability of clot time and clot development (α‐angle) values observed at site D. Clot times tended to be lower compared with sites A–C, which may be explained by the higher TF content masking the hypocoagulant state of haemophilia. At site D, the preanalytical conditions including inter‐operator experience preparing the diluted reagents were different compared with the other sites. The high inter‐site and intra‐site variability observed for site D impedes interpretation of the results and demonstrates the importance of standardised TF preparation and handling protocols to ensure reproducibility and comparability across studies and clinical sites.

Inhibition of the contact pathway by adding corn trypsin inhibitor (CTI) to the citrate tubes used for blood sampling could limit variability when low TF is used to initiate the assay; however, the SSC of the ISTH did not provide any recommendations on the use of CTI [6]. In the thrombin generation assay, the presence of CTI during blood sampling resulted in a reduction of peak thrombin and ETP in normal blood, but no major differences were seen with and without CTI in haemophilia A or B baseline samples, or in the concentration‐response curve for concizumab added to haemophilia plasma [21]. This is most likely a result of contact activation playing a limited role in the absence of FVIII or FIX. Similar results were seen for in vitro spiking of concizumab in ROTEM (data not shown). For this reason, and due to limited access and short shelf‐Iife of citrate tubes containing CTI, contact pathway inhibition was not employed in the present study.

There were two bleeding events reported during this study without any apparent influence on the ROTEM values obtained one day before or one day after the bleeds were recorded. However, due to their low number and the fact that the bleeds did not occur at the same time as the ROTEM measurements, it is not possible to make any firm conclusions about how bleeding episodes may affect ROTEM and its applicability in clinical settings.

Limitations of this study include the small number of patients (*n* = 6) in the final analysis, and that only four concizumab‐naïve samples were available, restricting the generalizability of our findings. In addition, the diluted TF reagent used as an activator in this study has not been standardised and validated for routine clinical use [3]. This limits potential applicability of the modified ROTEM in clinical settings. The lack of correlation between ROTEM parameters, concizumab exposure and free TFPI at clinical centres capable of running the modified ROTEM assay implies that the modified ROTEM method is currently not suitable for monitoring patients on concizumab prophylaxis. A larger study including a greater number of samples – particularly from patients with suboptimal concizumab levels – would be required for a more robust assessment of the assay's potential clinical utility.

Overall, the results from the present study demonstrate that there remains a need for a standardised clinical assay to monitor coagulation functionality of patients while on anti‐TFPI prophylaxis. Future research could attempt to validate a modified ROTEM assay in a broader cohort and in special situations such as in patients undergoing surgery.

## 5 Conclusion

Based on the data from the present study, we cannot recommend the modified ROTEM assay with diluted TF reagent for general monitoring of coagulation potential for patients on concizumab prophylaxis, as not all clinical centres are able to perform the modified ROTEM assay with diluted TF reagent reliably. The data obtained from specialised sites that can perform the modified ROTEM assay reliably are too limited to determine whether results from this modified assay can be used for general monitoring.

## Author Contributions

Conceived the study: Pratima Chowdary, Hermann Eichler, Victor Jiménez‐Yuste. Carried out experiments: Kasper Jensen, Hermann Eichler, Anne Riddell, Nora V. Butta, Cecilia Augustsson. Data analysis: Andrea Paramo‐Florencio, Marianne Kjalke, Kasper Jensen. Data interpretation: All authors. All authors contributed to drafting and review of the manuscript and approved the final version for submission.

## Funding

The study was sponsored by Novo Nordisk A/S.

## Ethics Statement

The present study used samples from patients participating in the explorer 7 and 8 trials. Both trials were conducted in accordance with the Declaration of Helsinki and the applicable International Council for Harmonisation of Technical Requirements for Pharmaceuticals for Human Use Guideline for Good Clinical Practice.

## Patient Consent Statement

For samples from the explorer7 and explorer8 clinical trials, informed consent was obtained from the participants or their caregiver.

## Conflicts of Interest

H. Eichler: grant/research support from Bayer, CSL Behring, Pfizer, consultant for Bayer, BioMarin, CSL Behring, Novo Nordisk, Pfizer, speaker bureau of Bayer, BioMarin, CSL Behring, Novo Nordisk, Pfizer, Sobi.

N.V. Butta: grant/research support from Novo Nordisk, Grifols, Takeda, paid instructor at Novo Nordisk, Novartis, Octapharma, Roche.

A. Riddell: nothing to declare.

C. Augustsson: grant/research support from Novo Nordisk.

M. Kjalke: employee and minor shareholder at Novo Nordisk.

K. Jensen: employee at Novo Nordisk at the time of the study.

A.P. Florencio: employee at Novo Nordisk.

J. Astermark: grant/research support from Shire, Sobi/Biogen, Bayer, Octapharma, CSL Behring, consultant at Takeda, Sobi, Pfizer, Bayer, Novo Nordisk, CSL Behring, Sanofi, Roche, Octapharma, BioMarin, speaker bureau of Takeda, Sobi, Pfizer, Bayer, Novo Nordisk, CSL Behring, Sanofi, Roche, Octapharma, BioMarin.

P. Chowdary: grant/research support from Bayer, CSL Behring, Freeline, Novo Nordisk, Pfizer, SOBI and Takeda, served on advisory boards for Apcintex, Bayer, Boehringer Ingelheim, CSL Behring, Chugai, Freeline, Metagenomics, Novo Nordisk, Pfizer, Roche, Sanofi, Spark, Sobi and Takeda.

V.J. Yuste: grant/research support from Pfizer, Shire, Novo Nordisk, Sobi, Octapharma, Grifols, consultant at CSL Behring, Sobi, Octapharma, Grifols, Pfizer, Shire, Novo Nordisk, Roche, Biomarin, Sanofi, travel support from Pfizer, Shire, Novo Nordisk, Octapharma, Grifols.

## Data Availability

Research data are not shared.
